# The relationship between shyness and short video addiction among college students: chain mediating role of social support and basic psychological needs

**DOI:** 10.3389/fpsyg.2026.1773800

**Published:** 2026-05-08

**Authors:** Xiaofei Bu, Xin Li, Bingzhi Li, Xuan Zhang

**Affiliations:** 1 School of Marxism, Xinjiang Normal University, Urumqi, China; 2 School of Political Science and Law, Xinjiang Normal University, Urumqi, China

**Keywords:** basic psychological needs, college students, short video addiction, shyness, social support

## Abstract

**Objectives:**

In contemporary society, shyness has become increasingly prevalent. Short video addiction, a distinct and highly prevalent form of internet overuse, remains underexplored in relation to shyness, despite established links between shyness and broader internet addiction. Accordingly, further investigation is needed to understand the mechanisms through which shyness influences short video addiction among college students. Therefore, this research aims to investigate the connection between the two, incorporating the variables of social support and basic psychological needs to examine their roles in this relationship.

**Methods:**

Convenience sampling was used to recruit 1,513 students at a college in western China. Data were collected using the Shyness Scale, Short Video Addiction Scale, Social Support Rating Scale, and Basic Psychological Needs Scale. Correlation analysis was performed using SPSS 26.0, and mediation effects were analyzed using the PROCESS macro (Model 6).

**Results:**

(1) Shyness was significantly positively associated with short video addiction. (2) Social support and basic psychological needs independently mediated the relationship between shyness and short video addiction. (3) Social support and basic psychological needs sequentially mediated the relationship between shyness and short video addiction.

**Conclusion:**

This study elucidates the mediating roles of social support and basic psychological needs in the association between shyness and short video addiction. Strengthening college students’ social support systems may enhance the fulfillment of their basic psychological needs, thereby reducing the risk of short video addiction. These findings highlight the importance of addressing both social and psychological factors in prevention and intervention efforts.

## 1 Introduction

Against the backdrop of increasingly sophisticated mobile internet infrastructure, short videos have rapidly emerged as one of the dominant media formats, exemplifying the evolution of information dissemination paradigms. They are profoundly reshaping people’s entertainment, learning, and lifestyles. By June 2025, China’s online video user base had grown to 1.085 billion. Notably, short video platforms alone accounted for 1.068 billion users, representing a staggering 95.1% of the total internet population and establishing this format as the leading online application. With the increasing prevalence of short video applications, concerns have been raised regarding their addictive nature, particularly the risks posed to college students. Due to their fragmented, diverse, and interactive nature, short videos resonate deeply with the contemporary college student demographic (Zhang et al., 2019). Consequently, this demographic exhibits exceptionally high user stickiness toward short video platforms, with some individuals even developing addictive behaviors (Wan et al., 2025). Short video addiction refers to a clinical concept characterized by compulsive use, in which individuals develop intense psychological cravings and behavioral dependencies, resulting in chronic preoccupation (Fu et al., 2024). Research indicates that short video addiction impairs social and daily functioning, including reduced physical exercise, disrupted sleep cycles, as well as heightened loneliness and depression (Qu et al., 2024).

As a relatively common personality trait (Sato et al., 2018), shyness refers to a state of discomfort or inhibition in social situations, characterized by apprehension of negative evaluation and accompanied by emotional distress or restraint (Chen, 2019; Hipson et al., 2019). High levels of shyness may exert multidimensional negative effects on college students’ cognition, emotions, and behavior, thereby impairing their academic performance and social functioning (Eggum-Wilkens et al., 2020; Teppers et al., 2014). Previous research has established an association between shyness and various forms of problematic internet use, including internet addiction and mobile phone addiction (Han et al., 2017; Li et al., 2023). Within this domain, short video addiction—the focus of the present study—is considered a specific subtype of internet addiction (Ye et al., 2022). However, despite being conceptually related to general internet addiction, short video addiction is believed to involve distinct psychological mechanisms due to the unique features of short video platforms (Hong et al., 2025). These platforms employ complex algorithms to deliver an endless stream of personalized content, which may trigger unique addictive processes. Moreover, short videos are characterized by their brevity, rapid pacing, and high-intensity stimulation, capable of eliciting strong dopaminergic responses within a very short timeframe. Given these distinctive characteristics, the mechanisms underlying short video addiction may differ from those implicated in general internet addiction. Although prior research has documented a link between shyness and general internet addiction, the specific pathways through which shyness influences short video addiction remain underexplored. To address this gap, the present study aims to investigate the relationship between shyness and short video addiction by examining the mediating roles of social support and basic psychological needs.

### 1.1 Shyness and short video addiction

Given that short video addiction constitutes a distinct manifestation of internet overuse, it is reasonable to infer that the mechanism by which shyness influences short video addiction may parallel that by which shyness affects general internet addiction. With the continuous advancement of the internet and smartphones technologies, research on the link between shyness and internet or smartphone addiction has been accumulating. Shy individuals constitute a high-risk group for excessive use of online technologies, as their susceptibility to negative emotions such as loneliness and depression serves as a key precursor driving the development of specific forms of internet addiction (Cheng et al., 2025), including short video addiction (Zhang L. et al., 2024; Zhao and Kou, 2024). Additionally, highly shy individuals typically exhibit elevated levels of immersion tendency. Immersion refers to a psychological state in which individuals feel enveloped by an environment and engages in continuous interaction with its steady flow of stimuli (Witmer and Singer, 1998). Although immersion tendency does not directly cause short video addiction, individuals with a high immersion tendency are more susceptible to experiencing immersion and pleasure when browsing short videos, and may turn to short videos as a means of escape and withdrawal. In summary, shyness is considered a significant influencing factor in short video addiction. Therefore, we propose Hypothesis 1: Shyness is significantly and positively associated with short video addiction.

### 1.2 The mediating role of social support

As a crucial interpersonal resource, social support comprises acquired social support and perceived social support (Liu et al., 2022). Existing research generally holds that, compared to objectively acquired practical support, subjectively perceived support has a more pronounced predictive effect on mental health (Uchino, 2004). Perceived social support refers to individuals’ subjective expectations and evaluations regarding the availability of support (Barrera, 1986). Functioning as a stable cognitive schema, perceived social support induces systematic interpretative biases: individuals with high levels tend to interpret ambiguous social cues as signals of support, whereas those with low levels are more likely to interpret such cues as signals of rejection or indifference (Brissette et al., 2002). Research indicates that shyness has a negative predictive effect on social support (Zhao et al., 2017). Shy individuals often encounter difficulties in establishing and maintaining close, satisfying relationships. Due to interpersonal communication challenges, they have fewer opportunities to interact with others, thereby obtaining less support from interpersonal connection. Furthermore, compared to non-shy individuals, shy individuals tend to be more passive in daily interactions, maintain fewer peer relationships, and experience lower levels of intimacy and support across various relationship contexts. With the widespread adoption of the internet and smartphones among college students, their sources of social support have diverged into online and offline pathways. Research indicates that these two channels exert contrasting effects on addictive behaviors: offline support serves as a significant protective factor against both internet addiction and smartphone addiction (Chang et al., 2022), with recent evidence confirming its negative predictive role for short video addiction as well (Yang et al., 2022); conversely, online support is associated with heightened risk (Wang and Wang, 2013). Given that shy individuals are more likely to experience deficits in offline social support, which may in turn increase their susceptibility to short video addiction, social support is proposed as a mediating mechanism in this relationship. Therefore, we propose Hypothesis 2: Social support mediates the relationship between shyness and short video addiction.

### 1.3 The mediating role of basic psychological needs

According to self-determination theory, satisfying the basic psychological needs for autonomy, competence, and relatedness is fundamental to achieving positive psychological development and behavioral self-regulation (Deci and Ryan, 2000). Although research directly examining the link between shyness and all three basic psychological needs remains limited, several studies have specifically explored its relationship with relatedness. Relatedness refers to the experience of warmth, connection, and care gained through establishing bonds with others and feeling significant to them (Pickett et al., 2004). When relatedness is thwarted, individuals perceive social distance, exclusion, and loneliness. Shy individuals, despite possessing a fundamental motivation to interact with others, often expend considerable time monitoring their own feelings and behaviors during social encounters, preoccupied with concerns about making a negative impression (Ray Crozier and Birdsey, 2003). Consequently, their need for relatedness frequently remains unfulfilled. More broadly, the frustration of basic psychological needs has been shown to activate maladaptive motivational processes, which in turn influence behavior and well-being (Vansteenkiste and Ryan, 2013). This theoretical framework helps explain the established link between unmet psychological needs and short video addiction. Short video platforms, through their algorithmic recommendation mechanisms, are adept at delivering sustained and potent gratification that precisely targets users’ basic psychological needs, constituting a core psychological pathway underpinning their addictive potential (Kardefelt-Winther, 2014). When individuals’ basic psychological needs are chronically unmet, they become particularly susceptible to the compensatory appeal of such platforms. Therefore, we propose Hypothesis 3: Basic psychological needs mediate the relationship between shyness and short video addiction.

### 1.4 The chain mediating role of social support and basic psychological needs

Previous research has consistently shown a positive link between social support and basic psychological needs (Wang, 2024). Social support derived from intimate relationships plays a pivotal part in fulfilling an individual’s needs for autonomy, competence, and relatedness (Deci and Ryan, 2000). Specifically, affective and informational support foster autonomy by creating environments that respect personal preferences, thereby encouraging decisions aligned with one’s values. Furthermore, by offering positive reinforcement and practical assistance, social support enhances individuals’ self-efficacy and resilience in tackling challenges, thereby elevating their perceived competence. Moreover, supportive interactions themselves establish stable, positive interpersonal bonds, directly fulfilling the fundamental need for relatedness. Due to interpersonal communication difficulties, shy individuals tend to perceive lower levels of social support compared to their non-shy counterparts, which in turn impairs the satisfaction of their basic psychological needs. This frustration of unmet needs subsequently heightens their vulnerability to short video addiction as a compensatory behavior. Therefore, we propose Hypothesis 4: Social support and basic psychological needs play a chain mediating role in the relationship between shyness and short video addiction.

## 2 Materials and methods

### 2.1 Participants

This study utilized data derived from an online survey of 1,513 students at a college in western China. Participants were selected using convenience sampling and completed the questionnaire anonymously and voluntarily. All participants were undergraduate students across all four academic years. The survey was administered via Wenjuanxing, an online questionnaire platform. Several lecturers were invited to distribute the questionnaire by sharing hyperlinks or QR codes with students. After data collection, invalid questionnaires were identified and excluded based on the following criteria: (a) incomplete responses (i.e., items left blank); (b) careless responses with obvious logical inconsistencies (e.g., contradictory answers to related questions); (c) failure to pass attention-check questions randomly inserted by the Wenjuanxing system; and (d) abnormally short or long response times (a reasonable response time was approximately 3–5 s per question). Following data cleaning, 75 invalid questionnaires were excluded, yielding 1,438 valid responses (an effective response rate of 95.0%). Among these valid samples, the mean age was 20.36 years (SD = 1.73). The demographic composition was as follows: 48.8% male and 51.2% female; the proportion of students in each year group (first to fourth year) was 38.3, 32.1, 18.6 and 11.0%, respectively.

### 2.2 Measures

#### 2.2.1 Shyness scale

The Revised Cheek and Buss Shyness Scale (RCBS) was employed to assess individuals’ shyness traits in this study (Cheek, 1983; Xiang et al., 2018), a measure demonstrating sound applicability among Chinese student populations. The scale comprises 13 items spanning two dimensions: shyness and sociability. In this study, the total score of the scale was used to represent the overall level of shyness, consistent with the theoretical focus on shyness as a global personality trait and with common practice in prior research (Chak and Leung, 2004). All items employed a 5-point Likert scale (1 = never, 5 = always), yielding a total score range of 13 to 65, where higher scores indicate stronger tendencies toward shyness. The Cronbach’s alpha coefficient for this scale in the present study was 0.94.

#### 2.2.2 Short video addiction scale

To ensure a validated assessment, this study employed Qin’s Short Video Addiction Scale for College Students (Qin, 2020). This 14-item scale encompasses four dimensions: withdrawal (5 items), avoidance (3 items), loss of control (4 items), and ineffectiveness (2 items). A 5-point Likert scale (1 = strongly disagree, 5 = strongly agree) was employed, with higher total scores indicating greater short video addiction tendencies. The Cronbach’s alpha coefficient for this scale in the present study was 0.90.

#### 2.2.3 Social support rating scale

The Social Support Rating Scale (SSRS) was designed by Shuiyuan Xiao based on the national context of China (Xiao, 1994). This assessment tool comprises 10 items, encompassing three dimensions: objective support (3 items), subjective support (4 items), and support utilization (3 items). The total scores range from 12 to 66. Greater levels of social support are indicated by higher scores. The Cronbach’s alpha coefficient for this scale in the present study was 0.71.

#### 2.2.4 Basic psychological needs scale

The study adopted the Chinese version of the General Scale of Satisfaction with Basic Needs (BNSG-S; Deci and Ryan, 2000; Liu et al., 2013). The scale comprises 21 items across three domains: autonomy needs (7 items), competence needs (6 items), and relatedness needs (8 items). Across the entire scale, there are 12 positively worded items and 9 negatively worded items, measured on a seven-point Likert scale ranging from 1 (not at all) to 7 (very much). Scores were calculated using the mean, with higher scores representing greater satisfaction. The Cronbach’s alpha coefficient for this scale in the present study was 0.72.

### 2.3 Data analysis

Descriptive statistics, correlation analysis, and common method bias testing were conducted using SPSS 26.0. To ensure the accuracy of the regression results, multicollinearity was assessed using the variance inflation factor (VIF). All VIF values were below the recommended threshold of 10, indicating no multicollinearity. All study variables were Z-score standardized prior to analysis to facilitate interpretation of the results. Mediation analysis was conducted using Model 6 of the PROCESS macro for SPSS (Hayes, 2013). To assess the significance of indirect effects, bias-corrected bootstrap 95% confidence intervals were generated using 5,000 bootstrap samples. An indirect effect was considered statistically significant if the bootstrap confidence interval did not include zero. All reported coefficients were standardized (*β*) to allow comparison of effect sizes across different paths. The significance level was set at *α* = 0.05.

## 3 Results

### 3.1 Common method bias test

As the study primarily employed questionnaire surveys for data collection, Harman’s single-factor test was conducted to assess common method bias. The results revealed three factors with eigenvalues greater than 1, with the first factor accounting for only 25.71% of the total variance—well below the 40% threshold. This suggests that common method bias did not pose a serious concern in this study.

### 3.2 Description and correlation

Correlation analysis indicated that shyness was significantly negatively correlated with social support and basic psychological needs, while significantly positively correlated with short video addiction. Short video addiction demonstrated a significant negative correlation with social support and basic psychological needs. Furthermore, social support exhibited a significant positive correlation with basic psychological needs (Table 1).

**Table 1:** Descriptive statistics and partial correlation analysis of variables (*n* = 1,438).

Variables	M	SD	1	2	3	4
Shyness	28.87	9.71	1			
Social support	62.67	10.99	−0.11***	1		
Basic psychological needs	96.83	13.27	−0.24***	0.13***	1	
Short video addiction	98.32	38.49	0.44***	−0.07***	−0.34***	1

***p < 0.001.

### 3.3 Analysis of the mediating effect

**Figure 1:**
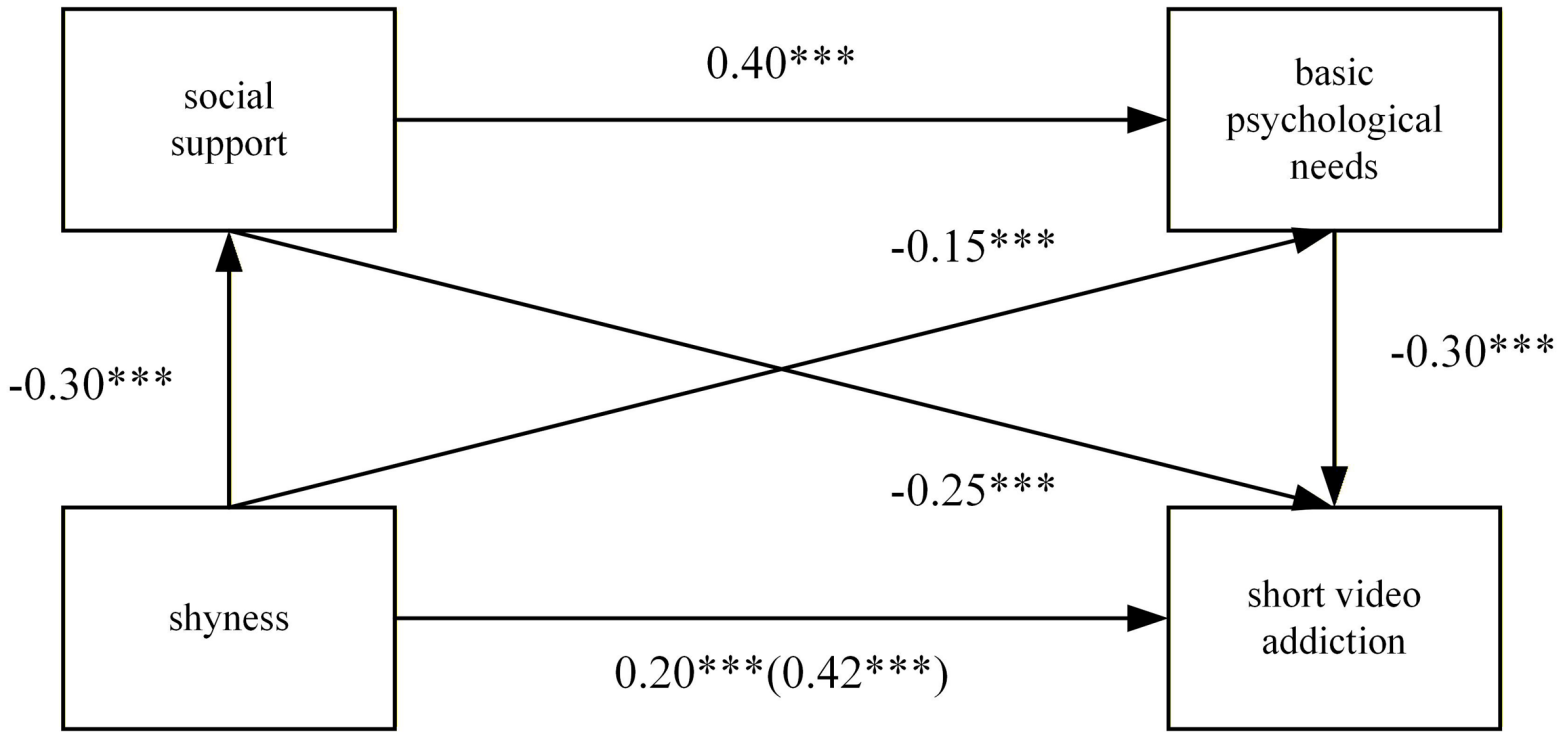
The chain mediation model. *p < 0.05, **p < 0.01, ***p < 0.001.

Multiple regression analysis was employed to test the hypotheses. As shown in Table 2, after controlling for student gender and grade, shyness significantly and positively predicted short video addiction (*β* = 0.42, *p* < 0.001). Shyness significantly negatively predicted social support (*β* = −0.03, *p* < 0.001) and basic psychological needs (*β* = −0.25, *p* < 0.001). Social support was a significant positive predictor of basic psychological needs (*β* = 0.40, *p* < 0.001). When all predictor variables were simultaneously included in the equation, shyness remained a significant positive predictor of short video addiction (*β* = 0.20, *p* < 0.001). Social support and basic psychological needs significantly negatively predicted short video addiction (*β* = −0.15, *p* < 0.001; *β* = −0.30, *p* < 0.001). These results indicate that the relationship between shyness and short video addiction was partially mediated by social support and basic psychological needs, providing support for Hypotheses 2 and 3. The predicted paths for each variable are illustrated in Figure 1.

**Table 2:** Regression results of the mediation model.

Regression equation		Overall fit index			Significance of regression coefficient	
Result variable	Predictive variable	*R*	*R^2^*	*F*	*β*	*t*
Short video addiction	Gender	0.43	0.19	108.67***	−0.03	−1.51
	Grade				0.02	0.91
	Shyness				0.42	20.33***
Social support	Gender	0.35	0.12	45.67***	0.10	2.05*
	Grade				−0.05	−1.00
	Shyness				−0.03	−8.50***
Basic psychological needs	Gender	0.45	0.20	55.43***	0.08	1.80
	Grade				−0.10	−2.20*
	Shyness				−0.25	−7.00***
	Social support				0.40	10.00***
Short video addiction	Gender	0.60	0.36	120.75***	0.12	3.00**
	Grade				−0.15	−3.75***
	Shyness				0.20	5.00***
	Social support				−0.15	−3.75***
	Basic psychological needs				−0.30	−7.50***

*p < 0.05, **p < 0.01, ***p < 0.001.

The analysis of mediating effects revealed that shyness exerted a significant direct effect on short video addiction, with a 95% confidence interval of [0.36, 0.48]. This interval did not contain zero, accounting for 61.76% of the total effect (*β* = 0.68; Table 3). Concurrently, the study identified three significant mediating pathways: first, a single mediating pathway for social support (*β* = 0.04), with a 95% CI of [0.01, 0.08] and an effect contribution of 5.67%; second, a single mediating path for basic psychological needs (*β* = 0.13), with a 95% CI of [0.09, 0.18] and an effect contribution of 18.41%; third, a chain mediating path for social support and basic psychological needs (*β* = 0.10), with a 95% CI of [0.07, 0.14] and an effect contribution of 14.16%. The confidence intervals for all mediating paths excluded zero, indicating statistically significant mediating effects. Overall, the mediating effect value *β* was 0.26, accounting for 38.24% of the total effect.

**Table 3:** Mediation effect tests according to bootstrap.

Path	Effect value	Boot SE	Bootstrap 95% CI	Proportion of relative effect
Total effect	0.68	0.02	[0.64, 0.72]	100.00%
Direct effect	0.42	0.03	[0.36, 0.48]	61.76%
Total indirect effect	0.26	0.02	[0.22, 0.30]	38.24%
Path 1: shyness→social support→short video addiction	0.04	0.02	[0.01, 0.08]	5.67%
Path 2: shyness→basic psychological needs→short video addiction	0.13	0.02	[0.09, 0.18]	18.41%
Path 3: shyness→social support→basic psychological needs→short video addiction	0.10	0.02	[0.07, 0.14]	14.16%

## 4 Discussion

### 4.1 Direct effect of shyness on short video addiction

This study found that shyness positively predicted short video addiction among college students, thereby validating Hypothesis 1 and supporting prior research findings (Ang et al., 2018). This finding is consistent with Triadic Reciprocal Determinism (Bandura, 1989), which posits that individual factors such as personality and cognition influence behavioral outcomes. Shy individuals, characterized by timidity and sensitivity, often experience heightened negative emotions such as fear and anxiety in real-life social situations (Firouzi et al., 2024; Wang and Zhao, 2026). These negative experiences may lead them to adopt maladaptive coping strategies, such as excessive short video use (Liu C. et al., 2025; Wang G. et al., 2025), as a means of emotional relief and situational escape. Furthermore, the user-friendly design and entertainment features of short video applications reinforce college students’ tendency toward excessive use (Zhang et al., 2026). Shy individuals also tend to exhibit poorer self-control (Li et al., 2022), making them more susceptible to losing control when using short videos to relieve stress, which may ultimately culminate in addictive behavior (Wang K. et al., 2025). This finding extends research on shyness as a risk factor for addiction from the domains of internet and mobile phone addiction to the emerging context of short video addiction, validating the consistent influence of shyness as a personality trait across different technological media (Bai et al., 2025; Satici, 2019). In the Chinese cultural context, where social harmony and interpersonal evaluation are highly valued (Fu, 2025), shy individuals may experience even greater social pressure, rendering the escapist appeal of short video platforms particularly strong.

### 4.2 Mediating effect of social support

This study found that social support mediated the relationship between shyness and short video addiction, thereby confirming Hypothesis 2 and supporting the findings of previous research (Gao et al., 2024). Consistent with prior studies, shy individuals tend to have lower levels of social support (Pan et al., 2024). Shy individuals often exhibit passive behavior in real-life interactions and maintain smaller interpersonal networks, resulting in limited perceived social support in daily life (Chan, 2012). When individuals receive low levels of social support, they are more susceptible to short video addiction (Ding et al., 2024). According to the cognitive-behavioral model (Davis, 2001), environmental pressures such as insufficient social support or social isolation in the real world may induce maladaptive cognitions in individuals, thereby prompting them to engage in compulsive internet use as a compensatory mechanism (Linh et al., 2026; Liu J. et al., 2025). Accordingly, the higher the level of shyness among college students, the lower their perceived social support (Hassan and Schmidt, 2023), which in turn elevates their risk of short video addiction (Liu et al., 2026). This finding elucidates a key mechanism linking shyness to addictive behavior: shyness does not directly cause addiction but instead undermines social support, depriving individuals of the environmental resources necessary for coping with stress and driving them to seek compensation in the virtual world. In Chinese universities, where dormitory life and peer relationships play a central role, the erosion of social support may be particularly detrimental (Xu et al., 2019), cutting students off from primary coping resources in this collectivist cultural setting.

### 4.3 Mediating effect of basic psychological needs

This study found that basic psychological needs mediated the relationship between shyness and short video addiction, thereby confirming Hypothesis 3 and supporting previous research (Gugliandolo et al., 2020). Shy individuals often adopt a passive stance in real-life interactions, struggling to present themselves confidently and share their thoughts (Wang and Zhao, 2026). In addition, they fear that their communication style may be inappropriate or that their verbal expression may be inadequate, leading to a lack of confidence in their own words and actions (Ime et al., 2024). These characteristics of shy individuals limit their ability to fulfill their needs for autonomy, competence, and relatedness in real-world environments (Mercader-Rubio et al., 2023). Furthermore, the satisfaction of college students’ basic psychological needs is closely linked to short video addiction (Kaya et al., 2024). According to Self-Determination Theory (Ryan and Deci, 2000), when individuals’ basic psychological needs are thwarted, they may turn to alternative environments and risk falling into a state of maladjustment. Short videos are characterized by rich information, immediate feedback, and ease of access. When an individual’s basic psychological needs are frustrated in real-life contexts, vivid and engaging short video content can rapidly alleviate negative emotions, providing a form of compensatory satisfaction (Zhang et al., 2025). This leads to immersion in short videos and may ultimately result in short video addiction. Consequently, shyness indirectly increases the risk of short video addiction through the mediating mechanism of reduced fulfillment of basic psychological needs (Zhang Y. et al., 2024). This finding extends the application of Self-Determination Theory from general internet addiction to the specific domain of short video addiction. Within the Chinese educational system, where academic pressure often limits opportunities for autonomy and competence (Li et al., 2026), the compensatory appeal of short video platforms may be especially pronounced for shy individuals.

### 4.4 Chain mediating effect of social support and basic psychological needs

This study found that social support and basic psychological needs exerted a chained mediating effect between shyness and short video addiction, thereby validating Hypothesis 4. Specifically, shyness negatively predicted social support among college students, which in turn predicted the satisfaction of basic psychological needs. When social support is insufficient, these basic psychological needs become frustrated (Wang, 2024). Ultimately, the frustration of basic psychological needs is closely associated with a tendency toward short video addiction, as individuals turn to algorithm-driven platforms to seek compensatory fulfillment (Karaman and Arslan, 2024), thereby completing the entire causal chain. The establishment of this serial mediation model holds significant theoretical importance. It reveals the sequential order and progressive relationship between environmental factors (social support) and intrinsic psychological factors (basic psychological needs) in the process by which personality traits influence behavior (La Guardia et al., 2000). This provides empirical support for the core proposition of Self-Determination Theory.

The mediation analysis revealed that the total indirect effect accounted for 38.24% of the total effect, while the direct effect accounted for the remaining 61.76%. This pattern of results carries several theoretical implications. The significant direct effect suggests that shyness may influence short video addiction through pathways beyond the two mediating mechanisms examined in this study. According to the I-PACE model, personality traits such as shyness may directly contribute to addictive behaviors through affective and cognitive responses that are not fully captured by social support and basic psychological needs. Future research could explore additional mediators, such as maladaptive cognitions or emotion regulation strategies, to further elucidate the mechanisms linking shyness to short video addiction. Moreover, the relative contributions of the three indirect pathways provide nuanced insights into the sequential mechanisms at play. Notably, the chain mediating pathway accounted for 14.16% of the total effect, highlighting the importance of considering the sequential interplay between environmental and psychological factors. This finding is consistent with Self-Determination Theory, which posits that social-contextual factors (such as social support) influence behavioral outcomes primarily through their impact on basic psychological need satisfaction. The observation that the chain mediating pathway explained a substantial proportion of the indirect effect underscores the critical role of this sequential mechanism.

This serial mediation model reveals the sequential and progressive relationship between environmental factors (social support) and intrinsic psychological factors (basic psychological needs) in the process by which personality traits influence behavior, thereby providing empirical support for the core proposition of Self-Determination Theory. In summary, by constructing a serial mediation model involving social support and basic psychological needs, this study provides an integrated theoretical framework for understanding the complex relationship between shyness and short video addiction (Sheldon and Niemiec, 2006). It highlights the importance of comprehensively considering personality traits, socio-environmental factors, psychological mechanisms, and their interrelationships when seeking a deeper understanding of the dynamics of technology addiction among vulnerable populations (Brand et al., 2016).

### 4.5 Limitations and future research

A number of methodological limitations in this study point to directions for improvement in future research. Firstly, the cross-sectional design imposes constraints on inferring causal relationships between variables. Although the theoretical model posits specific directional pathways, the associations between variables require validation through longitudinal studies or experimental interventions. Secondly, data collection relied entirely on self-reporting. For socially sensitive topics such as short video addiction, respondents may underreport actual circumstances due to social desirability bias, introducing a risk of systematic underestimation in measurement outcomes. Thirdly, although Harman’s single-factor test indicated that common method bias was not a significant concern in this study, this method has recognized limitations (Podsakoff et al., 2012). Future research could employ more rigorous techniques, such as the unmeasured latent method factor approach or multi-method data collection (e.g., combining self-reports with behavioral data or peer reports), to further mitigate potential common method bias. Finally, the sample was collected solely from college students, a cohort characterized by relative homogeneity in age and social environment. Consequently, the external validity of the findings is constrained, necessitating future validation of the model’s generalizability across broader and more heterogeneous social groups.

## 5 Conclusion

The study reveals a direct association between shyness and short video addiction, as well as an indirect association through a chain mediating pathway involving social support and basic psychological needs. This finding provides an integrated theoretical framework for understanding the psychological mechanisms underlying short video addiction among shy individuals, highlighting the critical sequential pathway from deficient social support to unfulfilled basic psychological needs. From a practical perspective, interventions aimed at strengthening social support and satisfying basic psychological needs constitute effective strategies for addressing or preventing short video addiction among college students. In parallel, psychological interventions targeting shyness itself may also reduce addiction risk at its root, thereby offering a multi-layered approach to intervention.

### 5.1 Implications

This study has both theoretical and practical implications. Theoretically, this study not only enriches the research in this field but also extends the application of Self-Determination Theory to the context of short video addiction, demonstrating that need frustration serves as a key mechanism linking personality traits to addictive behaviors. Practically, shy individuals are more susceptible to short video addiction due to their weaker social support networks and the consequent frustration of their basic psychological needs. To help such individuals overcome their dependence on short videos, two types of intervention should be implemented, namely strengthening social support and satisfying basic psychological needs. To strengthen social support, intervention programs could focus on creating opportunities for positive social interactions. Structured group activities, peer mentoring programs, and social skills training can help shy individuals build meaningful connections and enhance their perceived social support. Basic psychological needs can be satisfied by creating supportive environments—such as family counseling, teacher-student mentoring, and online support communities—that instill trust and help transform the irrational and negative beliefs shy individuals often hold about social relationships. By fulfilling these core needs, the compensatory appeal of algorithm-driven short video platforms may be significantly reduced.

## Data Availability

The original contributions presented in the study are included in the article/supplementary material, further inquiries can be directed to the corresponding author.
